# Implementation of the Community Component of the Mental Health Gap Action Programme (mhGAP): A Scoping Review

**DOI:** 10.3389/phrs.2025.1607759

**Published:** 2025-02-25

**Authors:** Felipe Agudelo-Hernández, Ana Belén Giraldo-Álvarez

**Affiliations:** ^1^ Facultad de Ciencias para la Salud, Universidad de Manizales, Manizales, Colombia; ^2^ Facultad de Ciencias para la Salud, Universidad de Caldas, Manizales, Colombia

**Keywords:** community mental health services, mental health recovery, community participation, implementation science, psychosocial support systems

## Abstract

**Objectives:**

To identify implementation variables and justify the use of the community component of the Mental Health Gap Action Programme (mhGAP).

**Method:**

The search was carried out in Cochrane, PubMed, Emerald, Scopus, Scielo, Redalyc and Google Scholar databases. Studies that analyzed the implementation of the community component of mhGAP were included, excluding those focused solely on the clinical component of mhGAP.

**Results:**

Out of the 726 records initially identified, only four met the inclusion criteria. The findings reveal that the evaluation of the community component of mhGAP has primarily been conducted in conjunction with other global and community mental health strategies, as part of multimodal approaches. Factors are recognized as key barriers and facilitators for the successful implementation of the program.

**Conclusion:**

The community component of mhGAP presents itself as a promising proposal to strengthen community-based mental health strategies. However, there is an urgent need to generate more evidence on the implementation of these strategies, particularly in terms of resource availability, long-term sustainability, and outcome monitoring.

## Introduction

In the Americas, there are significant mental health gaps. The prevalence of mental disorders and substance use disorders, which account for 10.5% of the global disease burden, contrasts with an average treatment gap of 65.7%. In Latin America and the Caribbean, this figure reaches 74.7%, surpassing 50% in children and adolescents [1, 2].

The main causes of this gap include insufficient investment in mental health services, weaknesses in the implementation of community models, and an excessive reliance on costly and ineffective hospital systems. Additionally, the lack of trained personnel, the low availability of essential medications, and the absence of mental health integration into primary care exacerbate the problem. Individual barriers such as stigma, distrust in the healthcare system, and low mental health literacy also limit access to care and treatment [1, 2].

To address these gaps, it is essential to strengthen health system capacity by reinforcing community-based mental health models and strategies, while promoting culturally adapted care. Furthermore, it is necessary to foster community education on mental health to reduce stigma.

Community participation in mental healthcare is critical for addressing access barriers, advancing a rights-based approach, and promoting a comprehensive perspective in this field. The implementation of community-based mental health strategies necessitates a broader understanding of health beyond traditional healthcare, consistent with the core principles of global mental health. This field emphasizes equity and is enriched by contributions from diverse disciplines, including health services research and implementation science [1, 2]. In 2018, the Lancet Commission on Global Mental Health and Sustainable Development put forward four pillars to address persistent gaps that require attention [3]: Mental health as a public good, mental health conditions as a continuum, the relationship of mental disorders with social and environmental influences, and health as a human right. In low- and middle-income countries, where the gap in mental health is greater, the above has been validated with tools such as the WHO Mental Health Action Plan 2013–2030 [4] or by the Sustainable Development Goals instead of 2030 Health Agenda [5].

The persistence of poverty, food insecurity and gender violence, as well as humanitarian crises derived from conflicts and climate change, exert a strong influence on the mental health of communities [6]. The strengthening of global mental health is based on the precept of equity, which requires the adaptation of different strategies and approach models, depending on the characteristics and resources that has each region [7]. For this, the evaluation from a focus on determinants and social, political and structural determination of health allows for the prioritization of actions that highlight the role of the community in mental healthcare [8].

Primary Healthcare (PHC) is considered essential to facilitate the population’s access to health promotion, disease prevention, care, treatment and rehabilitation services. In addition, it is recognized as a gateway to the health system and social care services, providing continuous and comprehensive care that addresses the health needs of people and communities [4].

Strategies proposed as essential to PHC include those that reduce costs and offer interventions to more people, in addition to strengthening community health and social medicine as theoretical foundations for contextualized care [9].

Similarly, decolonizing approaches to global mental health have gained momentum by proposing that interventions should be planned within local contexts to promote autonomy and agency in communities [10, 11].

In this regard, the World Health Organization (WHO) launched the Mental Health Gap Action Programme (mhGAP) in 2008 [12]. The mhGAP consists of a training and support program for different audiences, such as community leaders, healthcare personnel, governments and decision makers, mainly aimed at low- and middle-income countries [4, 9, 12, 13]. This program focuses on the evaluation and treatment of people with mental and neurological disorders [4, 9, 12, 13].

While there are different mhGAP intervention guides—namely, the mhGAP Intervention Guide (mhGAP-IG), the mhGAP Community Toolkit, and the mhGAP Humanitarian Intervention Guide (mhGAP-HIG)—there is significantly more evidence supporting the use of the clinical intervention guide [mhGAP Intervention Guide (mhGAP-IG)] [4, 12–14]. The community component of mhGAP is understood as the “mhGAP Community Toolkit” and those strategies that, although part of mhGAP-IG, specifically target communities without developing clinical components at the community level. Although this component has been used and recommended due to its potential to address mental health gaps, evidence on the outcomes of its use remains limited [15].

The mhGAP Tools for Community Use are part of the WHO’s Action Programme for Mental Health Gap (mhGAP), which aims for individuals with mental health disorders to receive high-quality, evidence-based mental health services, with a view to achieving universal health coverage. The purpose of these tools is to promote the expansion of mental health services beyond the primary healthcare setting. The mhGAP aims to enhance the participation of national and local authorities by allocating economic and professional resources to expand evidence-based mental health interventions.

In order to improve mental healthcare, the expansion of health services has been considered a central strategy that consists of four basic components: 1) primary healthcare services; 2) health education; 3) training of health workers; and 4) community management. These elements must be implemented alongside the strengthening of Primary Healthcare, seeking to combine efforts to ensure universal health coverage [16]. In this sense, the WHO’s Action Programme for Mental Health Gap (mhGAP) is based on guidelines, instruments, and training methods supported by scientific data to expand services in countries, especially in resource-limited settings [5, 9, 12].

Specifically, from a policy perspective, efforts have been made to ensure the reorganization and expansion of mental health services and systems. In this context, there is a push to integrate the updated WHO Mental Health Action Plan 2013–2030, with the aim of implementing more integrated approaches aligned with reducing gaps in mental healthcare. This effort was reinforced by the Sustainable Development Goals instead of 2030 Health Agenda.

### The Present Study

The mhGAP community toolkit was developed as a strategy aimed at promoting the expansion of mental health services that go beyond the primary healthcare setting. This objective is pursued through: 1. Identifying opportunities that exist within communities for mental health (prevention, promotion, and increasing access to mental health services); 2. Guiding the identification of local mental health needs by leveraging available resources and opportunities, as well as roles within the local community; 3. Providing recommendations for the development of activities, programs, and community-level mental health interventions; 4. Combating stigma, discrimination, social exclusion, and human rights abuses that affect people with mental health disorders.

In this context, the community component of mhGAP has the potential to operationalize approaches like Global Mental Health (GMH), which seeks to balance available mental health resources and reduce care gaps. This approach effectively addresses various implementation challenges arising from the diverse conditions across countries and regions. Consequently, it serves as a response to these challenges from a community perspective, influencing the social determinants of health and providing a pathway to tackle the significant and persistent gaps in care in a more contextualized manner.

The implementation of action strategies has been another significant challenge, as translating theories into practice requires a complex process. Implementation sciences facilitate bridging the gap between intervention planning and actual application, especially in economically constrained environments [17]. Therefore, identifying the core components and important implementation variables of the community component of mhGAP provides a valuable foundation for designing, implementing, and evaluating the community component of mhGAP t in diverse settings. Implementation sciences offer a comprehensive view of multi-level and interdisciplinary variables, emphasizing collaboration among research, policy, and implementation teams [17]. Thus, implementation sciences provide methodologies for the structured evaluation of these variables, allowing the synthesis of evidence regarding the use of the community component of mhGAP to yield an overview of common components in the processes already developed for its practical application.

However, despite a solid public health justification for the toolkit’s implementation and recommendations for its application, development, and adaptation to different contexts, specific implementation aspects have not been detailed. This could hinder the development of the community component of mhGAP and lead to the persistence of the mental health treatment gap, preventing the optimization of resources needed to close it. Therefore, the aim of this review was to establish the justification for implementing the community mhGAP program and to identify the variables in the implementation process.

## Methods

The present scoping review is based on the methodological framework presented by Arksey and O’Malley [18], as well as the methodology manual published by the Joanna Briggs Institute for Scoping Reviews. Scoping reviews aim to rapidly map the key concepts underpinning an area of research and the main sources and types of evidence available, and can be undertaken as stand-alone projects in their own right, especially when an area is complex or has not been thoroughly reviewed before [19]. Although mhGAP has been previously addressed in other reviews (particularly from its clinical component), the community component does not have a prior comprehensive analysis.

### Inclusion, Exclusion and Sample Criteria

To carry out the scoping review, the Cochrane, Pubmed, Emerald, Scopus, Scielo and Redalyc databases were used, and gray literature searches were also carried out. The Boolean code was selected from Mesh descriptors and definitions widely accepted in the literature, specifically for the term “Community Mental Health AND mhGAP.”

The research was carried out using the following keywords (Mesh and desc): [(Community Mental Health Centers OR Community Supports OR Community Resources OR Community Integration OR Community Health Services OR Community Networks OR Community Participation) AND mhGAP AND (implementation science OR implementation)], in the aforementioned databases. Additionally, manual searches of the reference lists of relevant articles were carried out to identify those that were not generated in the database search.

We conducted the search between 2008 and 2024, as mhGAP implementation began globally in 2008. The selected studies had to meet the following inclusion criteria: original studies, reviews or meta-analyses that report on the need to implement mhGAP, its development, especially in the community component. Studies describing only aspects of clinical mhGAP were not included. A total of 20 studies that only described the implementation of the clinical component of the mhGAP were excluded.

### Study Protocol

The steps recommended by the Joanna Briggs Institute, in addition to those proposed by Arksey and O'Malley [18], were followed, using the PCC [Population, Concept and Context] strategy to formulate the review question. The PCC descriptors (Population, Concept, and Context) are used to structure the review questions in a Scoping Review. In this case, “P” refers to the Population, which includes the individuals or institutions that utilize or receive support within the framework of the community-based mhGAP. “C” represents the Concept, focused on the core components justifying the implementation of the community-based mhGAP and the implementation variables required for its effective application. The second “C” refers to the Context, which encompasses the community interventions of the mhGAP and the social conditions influencing the program’s implementation. The use of these descriptors facilitated the formulation of clearer and more specific questions, thereby streamlining the analysis.

The review questions were: “What is the need to implement the community component of the mhGAP? What are the implementation variables or characteristics?.” The question refers to the justification for implementing mhGAP, including the gaps for its implementation.

After performing the search, bibliographic citations were identified in the EndNote X9/2018 program, and duplicate studies were eliminated. For study selection, two researchers initially reviewed the titles and abstracts according to the inclusion criteria. Verification of the eligibility criteria was carried out using a random sample of 25 articles. Using Cohen’s K coefficient, agreement between observers was determined, which was 0.86 (95% CI: 0.66–1.00), considered good agreement.

The selection of studies was carried out by consensus of the panel of reviewers, in accordance with the critical appraisal tools, and those that passed the quality assessment were included in the review.

The Newcastle-Ottawa Scale [20] was used to assess the quality of non-randomized studies in this study. This tool evaluates three key dimensions: the selection of study groups, the comparability of the groups, and the determination of exposure or the outcome of interest. To ensure the quality of the included studies, compliance with the inclusion criteria was specified, which helped address the risk of bias and ensure the validity and relevance of the findings in this scoping review. They were considered of acceptable quality when the four evaluators agreed that 70% of the elements of the evaluation instruments were positive. Two reviewers with a doctorate in social sciences and a master’s degree in community psychology evaluated titles and abstracts according to the inclusion and exclusion criteria. Following this, the full text was reviewed for eligibility.

All eligible articles were entered into Microsoft Excel, where the following information was extracted: publication type, study objectives, the community component of mhGAP background. There are also included: Community participation method, type of study, sample, mental health diagnoses, life course of the sample, results, description of the improvement indicator. Implementation variables include: acceptability, adoptability, suitability, feasibility, fidelity, implementation cost, penetration and sustainability.

In addition, the method of Tricco et al. [20, 21] was used, with the following steps: 1) Identify the research question by clarifying and linking the purpose and research question, 2) identify relevant studies by balancing feasibility with breadth and comprehensiveness, 3) select studies using an iterative team approach to study selection and data extraction, 4) represent the data incorporating a numerical summary and qualitative thematic analysis, 5) collate, summarize, and report the results, including implications for policy, practice, or research, and 6) consultation exercise, where we considered public policy documents given the limited information on the community component. In this sense, data were organized and analyzed using a conventional content analysis approach, referencing research questions as a guide. Duplicate components or features were removed. If some search results fell into multiple categories, agreement was sought with the researchers.

## Results

**FIGURE 1:**
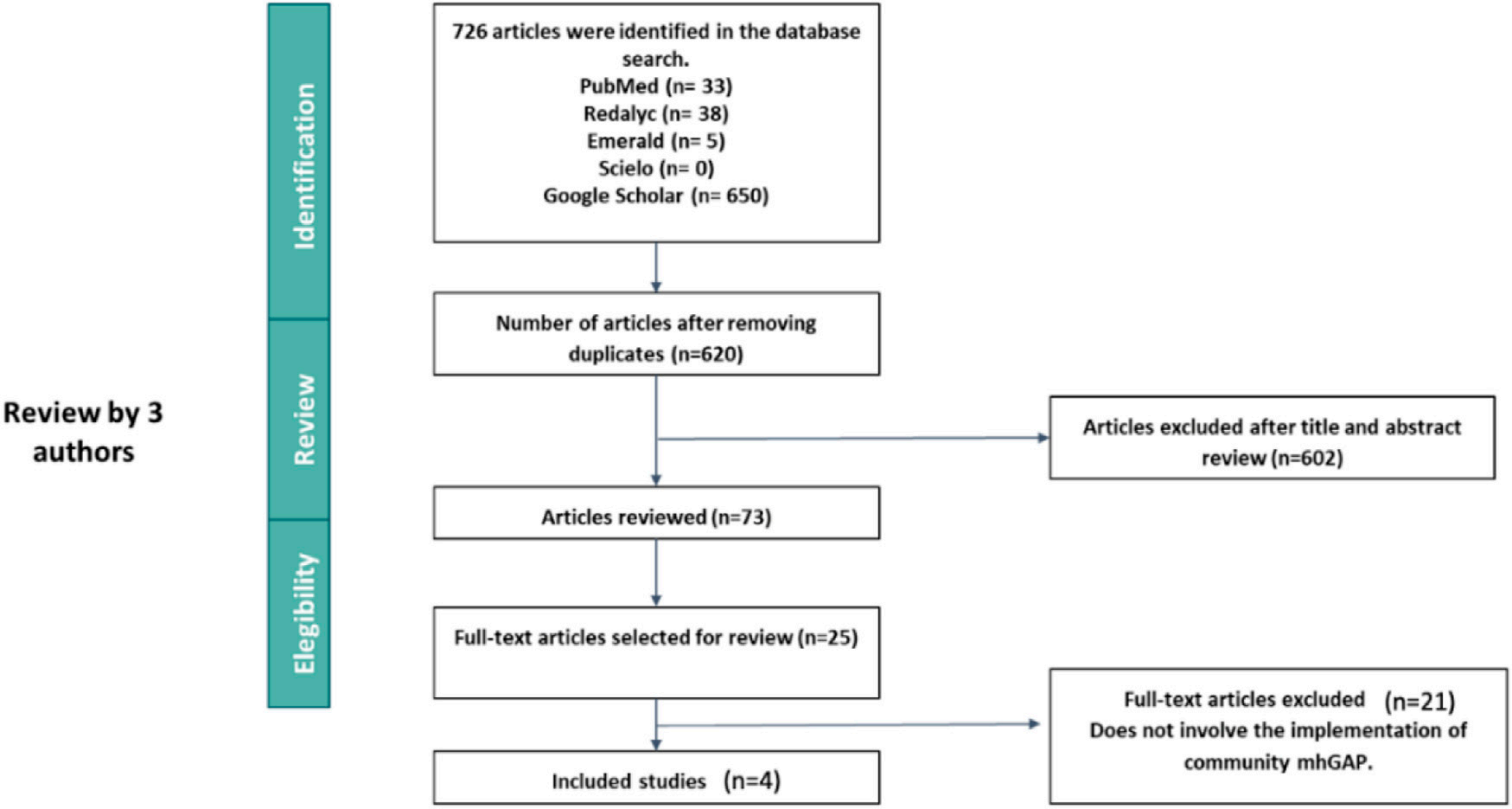
Results of implementation of the community component of the Mental Health Gap Action Programme (mhGAP): A scoping review, from 2008 to 2024 (worldwide).

Four articles met the inclusion criteria for the present Scoping review (Figure 1).

Of the four articles included, the majority correspond to studies carried out in low- and middle-income countries, with predominant narrative and cross-sectional review methodology as described in the table (Table 1). There are proposed as main categories: *The need to implement the* community component of mhGAP *and community component of the mhGAP implementation variables*.

**TABLE 1:** Implementation of the community component of the Mental Health Gap Action Programme (mhGAP): A scoping review, from 2008 to 2024 (worldwide).

Authors	Country	Study design	Aim	Use of the component of the mhGAP
Agudelo-Hernández et al. [16]	Colombia	Quasi-experimental	Demonstrated that people at risk of suicide who participated in this component experienced a greater reduction in psychosocial disability, compared to those who only received psychiatric care or participated only in the clinical component of the program	Elements from the different mhGAP programs are included to evaluate implementation variables, barriers, and facilitators
Hamdani et al. [22]	Pakistan	Two-arm, single-blind, hybrid, effectiveness implementation, cluster randomized controlled trial	To evaluate the effectiveness and scale-up implementation of the locally adapted WHO PST program delivered by volunteer families in rural Pakistan	mhGAP was used as a concomitant strategy with PST in order to enhance treatment
Lasisi et al. [23]	Nigeria	Randomized controlled trial with intervention and waiting list control groups.	To evaluate the effect of ADHD training program on the knowledge and attitudes of primary school teachers in Kaduna, Nigeria	To evaluate the effect of ADHD training program on the knowledge and attitudes of primary school teachers in Kaduna, Nigeria
Faregh et al. [24]	The projects were carried out in multiple areas of six countries (Chad, Ethiopia, Nigeria, Guinea and Haiti)	Informal consultation approach to analyze the authors’ combined field experience in the practice of mhGAP implementation and training	Explore key cultural and ethical dimensions of mhGAP planning, adaptation, training and implementation [1]. Concepts of wellbeing and illness: how to examine cultural norms, knowledge, values and attitudes in relation to “mhGAP culture” [2] Systems of care: identify formal and informal systems of care in the cultural context of practice [3] Ethical space: examine issues related to power dynamics, communication and decision making. Systematic consideration of these issues can guide the integration of cultural knowledge, structural competence, and ethics into implementation efforts	Proposal for cultural and contextual adaptations that can be applied when implementing mhGAP.

The authors.

The need to implement the community component of mhGAP.

### Recognition of the Importance of the Social Determinants of Mental Health

Among the results of this search, a Quasi-experimental study in Colombia that implemented the community component of mhGAP was identified, which demonstrated that people at risk of suicide who participated in this component experienced a greater reduction in psychosocial disability, compared to those who only received psychiatric care or participated only in the clinical component of the program [15]. There they found that many of the barriers to the adequate implementation of the strategies were related to the social, cultural and political conditions of the context such as stigma, the lack of solid support networks, geographical distances, armed conflict, administrative obstacles, among others. More broadly, the study found that the community-based mhGAP allows for the integration of community participation with healthcare to ensure a more comprehensive and contextualized approach for individuals at risk of suicide.

### Community Education as a Bridge to Stigma Reduction

Hamdani et al. [22] developed an integrated service delivery model that fuses social, technological, and business innovations targeting children with developmental disorders and delays in low-resource contexts. In order to provide standardized care, a mobile application was designed that incorporates the WHO mhGAP-IG diagnostic and management guidelines, as well as the WHO Parent Skills Training (PST). Although this integration did not exclusively address the community component of the mhGAP, it did incorporate educational and clinical strategies in the parent and family training process, led by community actors. Also, in addition to evaluating the impact of the interventions in relation to the impact on stigma and understanding of psychiatric conditions.

Educational interventions have been an aspect highlighted in the literature based on the use of mhGAP. In a randomized controlled trial carried out by Lasisi et al. [23], the impact of a training program on Attention Deficit Hyperactivity Disorder (ADHD) on the knowledge and attitudes of primary school teachers in Kaduna, Nigeria was evaluated. The educational strategy implemented was based on the ADHD component of the mhGAP. 85 teachers participated in the intervention group and 75 in the control group. After the intervention, there was a significant improvement in knowledge related to ADHD, a decrease in the negative perception of this diagnosis, and an increase in knowledge scores about behavioral interventions to address ADHD in the school setting.

Although the other two studies did not focus on evaluating the educational components of the tool, they do highlight the importance of the strategy as a clear and accessible training methodology that allows for adaptation to the needs of different communities.

### Cultural and Contextual Adaptation of Interventions

Faregh et al. [24] presented systematic reflections and *post hoc* analyzes based on the authors’ informal observations during their field experiences in mhGAP-related projects at various sites. Based on intensive field work experience, focused on capacity development, different challenges were identified. Among them were the cultural differences that impacted the ways of seeing and understanding mental disorders, the configuration of the local healthcare system, the level of prior knowledge and skills of the students, the sociopolitical context, among others. In order to address these problems, it is proposed to carry out processes of cultural and contextual adaptation of the different activities, promote the participation of the community and sectors involved, and the implementation of innovative and accompaniment processes that seek the continuity of the interventions.

In this context, they concluded that mental health training, although essential, does not automatically guarantee the desired results, so one must be open to new strategies and innovations that respect culture and context [24]. This requires the participation and commitment of various stakeholders, recognizing their ideas and needs.

Agudelo et al. emphasize that the implementation guide for the community component of mhGAP is determined by the functioning of the context. The various studies agree on the importance of assessing the conditions and the community in which the program will be implemented in order to identify the needs and resources of the environment and make use of them.

### Implementation Variables

None of the included studies evaluated exclusive component of the mhGAP implementation variables. Only the study published by Agudelo-Hernández et al. [15] identified implementation variables themselves for different mhGAP modalities. In the other studies, although no implementation variables were identified, some common elements were recognized that were seen as important factors in the implementation of mhGAP, generally added to other types of clinical and community interventions. These factors were found to be presented mainly as barriers to the implementation of comprehensive processes for addressing mental health in low-resource environments, and are also proposed as elements that could be mitigated to the extent that development conditions are improved in accordance with the needs and possibilities of the person. These elements are described in Table 2, and it is specified whether they are met in the included studies (Table 2).

**TABLE 2:** Implementation variables highlighted in studies from the scoping review on the implementation of the community component of the Mental Health Gap Action Programme from 2008 to 2024 (worldwide).

Author	Availability of the strategy	Accessibility	Contextual adaptation and acceptability	Training, supervision	Monitoring, sustainability
Agudelo-Hernández et al. [16]	X	XX	XX	XX	XXX
Hamdani et al. [22]	X	X	X	XXX	XX
Lasisi et al. [23]	X	X	--	XXX	--
Faregh et al. [24]	X	XX	XXX	XX	XX

The authors.

For their part, Agudelo-Hernández et al. [15] focused their efforts on recognizing barriers and facilitators associated with the implementation of mhGAP at the clinical and community level associated with implementation variables. They conclude that the implementation of intersectoral recovery routes, built with and for the territory, must be approached as an integrative strategy that allows responding to diverse and dynamic challenges that affect the mental health and wellbeing of people and communities [16].

Regarding challenges, Hamdani et al. [22] identified that vulnerable health systems, lack of financing, and shortages of trained health personnel persist as significant barriers to the development, expansion, and sustainability of these interventions. When considering these obstacles from another perspective, they could constitute key elements for the implementation of public mental health strategies in low-resource settings. Stigma reduction can be taken as a factor that arises in relation to the implementation of mhGAP-based educational programs. This is because they allow for improving knowledge and attitudes in crucial contexts for the healthy development of daily life, mainly in relation to children when it comes to diagnoses such as ADHD [23].

Mutiso et al. [25] by addressing the gap in mental health treatment in Kenya in their study, led to proposing, with the community, elements that can allow better integration of mhGAP into community interventions. This is how they recognized as a key element the fact of placing the person and the community at the center from the autonomous exercise of their agency capacity.

The studies refer to factors that impact the implementation process of the strategy, such as availability and access, including the various resources required for its proper development; adaptation to the context and culture, assessing acceptability and usability; and a comprehensive, flexible training process focused on the community’s needs and connected to the realities of the territory (Table 2).

## Discussion

In response to the need for consolidated strategies to reduce the mental health gap within a Primary Healthcare framework, this study aimed to establish the key aspects that justify the community component of the mhGAP and to identify elements of its implementation process. A scoping review was conducted rather than a systematic review for the purpose of identifying knowledge gaps, covering a body of literature to clarify concepts related to community-based mhGAP. These reviews can also be useful precursors to systematic reviews and can be used to confirm the relevance of inclusion criteria and potential questions [26]. In this case, the questions in the following review focus on features or concepts in papers or studies, and mapping, reporting or discussing these features of the strategy for implementation.

Throughout the research, it was observed that there are no specific evaluations regarding the community mhGAP. Instead, the existing literature focuses on interventions that combine various mental health tools at the clinical and community levels [15, 22–25]. This finding highlights the scarcity of specific research on the implementation of the community mhGAP, although multimodal approaches have been identified as a key strategy for improving public mental health [15–17, 22–25].

Some articles that were not included in this review, although relevant, provide useful recommendations for the development of mhGAP strategies in community contexts. For example, Mutiso et al. [25], addressing the treatment gap in mental health in Kenya, propose focusing interventions on the person and the community, fostering their capacity for agency. Rebello et al. [27] suggest strategies to improve community mental health, such as integrating mental health services into primary care, sharing tasks from intersectoral approaches, and training non-specialized personnel. Additionally, they emphasize the use of technological tools as means to improve access to services and reduce costs. These interventions seek to reduce stigma and expand the capacity of mental health systems.

Patel et al. [3] also identifies key strategies to close the treatment gap, such as involving trained lay professionals and advocating for the participation of people with mental disorders in defending their rights. Wenceslau and Ortega [28], in their theoretical analysis on the integration of mental health within primary care, propose the need to optimize resources in low-income regions where specialized care is not continuous. This approach aligns with the implementation variables identified in this study, which underline the importance of adapting mental health interventions to the local context and optimizing available resources.

In relation to strategies aimed at communities, training in clinical mhGAP has also proven crucial to closing gaps in availability and quality of care [29]. Keynejad et al. [30] conducted a systematic review to identify the evidence generated from its implementation. Preventive measures adopted by the community are highlighted as elements of great value, as well as ongoing community support linked to clinical follow-up [31].

Just as in the community component of the mhGAP addressed in this review, Keynejad et al. [30] identify significant implementation gaps at the clinical level and conclude that it is necessary to integrate primary care approaches with community care. Other proposals have pointed out the need for ongoing contact between health professionals and individuals, in addition to involving the family, community, and cultural sphere [30].

A strength of the community mhGAP is its ability to overcome approaches that promote homogenization in the understanding and management of mental health. Contextualization and cultural adaptation of interventions are fundamental for their effective implementation [4, 13]. Clinical implementation studies of the mhGAP have also concluded that it is necessary to adapt its application to the specific context in which it is intended to be used [16], as such adaptation is essential to ensure the relevance, acceptance, and continuity of interventions [32].

Cultural and contextual adaptation, meaningful community participation, and continuous follow-up are crucial for the acceptability, effectiveness, and sustainability of the program [17, 27]. The implementation of the mhGAP should provide a clear framework for integrating culture and addressing the needs of the population [17]. Approaches that integrate cultural adaptation, community participation, and ongoing support are proposed as the most effective for addressing mental health in primary care in low- and middle-income settings. Although evidence on the community mhGAP is still limited, these findings highlight the urgency of developing implementation strategies that are culturally relevant and sustainable, ensuring that mental health interventions adequately respond to the needs of the populations they target.

The four analyzed articles emphasize the importance of prioritizing the local customs and practices of the group being intervened. They recommend providing more concrete information about the roles of each involved actor, ensuring that everyone understands their responsibilities in the process. Furthermore, they highlight the need to train various actors in the different components of mhGAP, stressing that professionals should act as human beings rather than merely as health resources. This approach aims for each actor to have a clear understanding of what mhGAP entails in their community and how they can effectively apply it in their specific context.

No studies were found that applied specific implementation science frameworks in the context of mhGAP, nor were there any that demonstrated a clear approach from this perspective. Implementation science is essential for translating scientific evidence into effective health practices that adapt to local characteristics.

This would allow for a more precise evaluation of interventions, facilitating the identification of barriers and facilitators in their adoption, and offering concrete recommendations to improve their impact. However, its application in mhGAP and other mental health strategies remains limited, particularly in resource-constrained regions, underscoring the urgent need for further studies to address these gaps.

It has been noted that the implementation of mhGAP tends to follow a biomedical approach that does not adequately consider local needs and contexts. Many of the interventions associated with mhGAP have been developed in Europe and North America, raising questions about their effectiveness in other settings. Therefore, contextualization and cultural adaptation are essential elements to ensure that interventions are relevant and effective across diverse communities [16, 21, 33].

This review highlights the limited evidence supporting the use of specific community mhGAP tools. Nevertheless, it emphasizes the importance of integrating multimodal approaches that do not separate from clinical strategies and intersectoral interventions, aligning with mhGAP principles.

The implementation of the community component of mhGAP in countries with gaps in care is crucial to engage communities in a comprehensive approach to mental health. It helps reduce stigma, strengthens mental health governance, and fosters collaboration between health systems, communities, and other sectors. Moreover, it allows addressing barriers to treatment access and enhances culturally sensitive psychosocial recovery strategies.

### Limitations

Among the limitations of this study, it is acknowledged that the articles included primarily focused on the community component of mhGAP. This focus may have led to the exclusion of other relevant articles concerning the community component of mhGAP and other components recommended by the WHO. Specific mentions of community mhGAP were scarce, except for the study by Agudelo-Hernández et al. [16]. This underscores the need to broaden the search in grey literature, particularly for reports from health organizations that are implementing this component.

While the articles were carefully analyzed to ensure that those including any community component of the mhGAP were not excluded (according to the operational definition), it remains a limitation that some reviewed studies primarily focused on the clinical aspect of the mhGAP. These studies may have contained relevant components for the review’s objectives. Despite refining the search strategy and reviewing it with multiple researchers, it is possible that some terms were overlooked or not entirely precise.

The above highlights the need to continue strengthening knowledge management and the dissemination of demonstrative experiences in the local implementation of the practices proposed in the global policy. Although it was not possible to define the core components or the methodologies to be included in the study according to the criteria, it was found that countries such as Colombia [34], Paraguay [35], Argentina [36], and Costa Rica [37] are implementing this strategy.

The creation of repositories and records of good practices in the development of the mhGAP program is proposed, aiming to have clear information about the development process and its results. This can facilitate the systematization of experiences in public policies, both from the perspective of implementers and actors in the health system and the community.

It is essential to integrate research findings in the field of community mental health, ensuring that evidence is accessible and usable to inform the generation of effective policies and practices. Doing so strengthens the responsiveness of health systems to current challenges, thereby improving the quality and scope of services offered and making transversal approaches to recovery effective.

Additionally, it is crucial to consider the barriers and facilitators in the implementation process. The effective development of mental health initiatives often necessitates significant social, economic, and sometimes political changes, which are challenging to achieve without political will and sufficient resources. Future research should explore these aspects of community mhGAP, particularly through technical reports from various countries, and consider longitudinal studies to define implementation variables and assess their impact on individuals and health systems.

The study’s potential biases include the omission of relevant articles due to the selection of databases and search terms used. Additionally, the exclusion of studies focusing solely on the clinical component of mhGAP may have limited the information gathered, as some of these studies might contain valuable contextual insights. The lack of geographical diversity also represents a bias, as the included studies mainly originate from Western contexts, potentially excluding relevant studies from other regions.

Among the limitations of this study, the lack of exhaustive analysis on the scalability and cultural adaptation of mhGAP stands out. These aspects may present challenges for its implementation in low-resource settings. Future research should address these barriers to improve the applicability and effectiveness of the program.

In conclusion, the review of the evidence regarding the community component of mhGAP reveals a significant lack of information about its implementation. Nonetheless, it highlights its potential to promote a more contextualized approach that addresses local needs and resources, particularly in low- and middle-income countries.

To enhance the effectiveness of interventions, it is crucial to consider the specific realities and capacities of each community. Factors such as accessibility to services, cultural adaptation of interventions, ongoing training for personnel, and the accompaniment and follow-up of initiatives are essential for developing sustainable and effective mental health programs.

These findings underscore the urgent need to intensify research on the outcomes of mhGAP community strategies. Through rigorous evaluations and contextual adaptations, it will be possible to identify best practices that can make these interventions more relevant and effective in diverse settings.

## Abbreviations

mhGAP, Health Gap Action Programme; PHC, Primary Healthcare; WHO, World Health Organization; GMH, Global Mental Health; PCC, Population, Concept and Context.
